# Cross-sectional Comparison of Disparities by Race Using White vs Hispanic as Reference Among Children and Youths With Developmental Disabilities Referred for Speech Therapy

**DOI:** 10.1001/jamanetworkopen.2022.34453

**Published:** 2022-10-04

**Authors:** Thomas Elliott, Kortney Floyd James, Karen J. Coleman, Kia Skrine Jeffers, Claudia L. Nau, Kristen Choi

**Affiliations:** 1 Division of General Internal Medicine & Health Services Research, Department of Medicine, David Geffen School of Medicine, University of California, Los Angeles; 2 Department of Research & Evaluation, Kaiser Permanente Southern California, Pasadena; 3 Southern California Permanente Medical Group, Kaiser Permanente Southern California, Pasadena; 4 School of Nursing, University of California, Los Angeles; 5 Department of Health Policy and Management, Fielding School of Public Health, University of California, Los Angeles

## Abstract

**Importance:**

Health care research on racial disparities among children and youths has historically used the White race as a reference category with which other racial and ethnic groups are compared, which may inadvertently set up Whiteness as a standard for health.

**Objective:**

To compare 2 interpretations of an analysis of racial disparities in speech therapy receipt among children and youths with developmental disabilities: a traditional, White-referenced analysis and a Hispanic majority-referenced analysis.

**Design, Setting, and Participants:**

This cross-sectional study used multiple logistic regression to analyze speech therapy referrals for children, adolescents, and transition age youths in an integrated health care system in Southern California from 2017 to 2020. Eligible participants were children and youths up to age 26 years with 1 or more diagnosed intellectual or developmental disability (eg, autism spectrum disorder, speech or language delay, developmental delay, Down syndrome, and others).

**Exposures:**

Child or youth race and ethnicity as reported by parents or caregivers (Asian, Black and African American, Hispanic and Latinx, American Indian or Alaskan Native, Native Hawaiian or Pacific Islander, White, multiple, and other).

**Main Outcomes and Measures:**

Receipt of speech therapy within 1 year of referral.

**Results:**

A total 66 402 referrals were included; 65 833 referrals (99.1%) were for children under age 17 years, 47 323 (71.3%) were for boys, and 39 959 (60.2%) were commercially insured. A majority of participants were identified as Hispanic (36 705 [55.3%]); 6167 (9.3%) were identified as Asian, 4810 (7.2%) as Black, and 14 951 (22.5%) as White. In the traditional racial disparities model where the reference category was White, referrals of children and youths who identified as Hispanic, Black, Pacific Islander, and other had lower odds of actual receipt of speech therapy compared with referrals for White children and youths (Hispanic: OR, 0.79; 95% CI, 0.75-0.83; Black: OR, 0.72; 95% CI, 0.66-0.78; Pacific Islander: OR, 0.74; 95% CI, 0.57-0.98). When using the majority race group (Hispanic) as the reference category, referrals for children and youths who identified as White (OR, 1.26; 95% CI, 1.20-1.30), Asian (OR, 1.21; 95% CI, 1.12-1.30), and multiracial (OR, 1.35; 95% CI, 1.08-1.71) had higher odds of resulting in actual service receipt in comparison with referrals for Hispanic children and youths.

**Conclusions and Relevance:**

The cross-sectional study demonstrates the value of decentering Whiteness in interpreting racial disparities research and considering racial differences against multiple referents. Racial disparities researchers should consider investigating multiple between-group differences instead of exclusively using White as the default reference category.

## Key Points

**Question:**

Among children and youths with developmental disabilities referred for speech therapy, does the interpretation of study results change when the reference category is Hispanic rather than White?

**Findings:**

In this cross-sectional study with 66 402 speech therapy referrals, Hispanic, Black, and Pacific Islander children and youths as well as those from other minoritized racial and ethnic groups had lower odds of speech therapy referrals when White children and youths were the reference group. When using Hispanic as a reference, racial advantage for those who identified as White and Asian became more apparent, while racial disadvantage for those who identified as Black remained.

**Meaning:**

These results suggest that using multiple reference groups to interpret race disparities may result in greater sensitivity to health equity.

## Teaser

This cross-sectional study compares rates at which children with developmental disabilities referred to speech therapy received therapy by race and ethnicity.

## Introduction

Health care research on racial disparities has historically used the White race as a reference category with which all other racial and ethnic groups are compared.^1,2^ Although such research is ostensibly intended to identify disparities in health care access, utilization, or outcomes among minoritized racial and ethnic group patients so that disparities can be resolved, quantifying disparities in relation to the health of people who are White may inadvertently set up Whiteness as a norm or standard for health, even though it is not necessarily the case that ideal health is found in the White population.^3,4^ A preponderance of studies on racial disparities in health focuses on comparing people who belong to minoritized racial and ethnic groups with people who are White without explanation—even when White individuals do not make up the majority of the study population.^5,6,7,8^ This is despite recent studies demonstrating that there may be more heterogeneity in health within White-identified populations than there is between White individuals and members of other racial or ethnic groups.^9^ The prevailing approach to racial and ethnic disparities research conceptualizing the White race as the standard for ideal health has led to an overemphasis on race deficits, rather than racism, as the source of racial disparities in health care.^10,11,12,13,14^

The challenges with current approaches to racial disparities research are evident in child and youth health, especially among children with autism and developmental disabilities.^15^ Developmental disabilities are lifelong conditions of impairment in cognition, behavior, communication, or neurological systems (eg, autism spectrum disorder, Down syndrome) that often require regular general and specialty health services.^16,17^ Black and Hispanic children have been found to be less likely to receive specialty behavioral health services, individualized education plans, speech therapy, occupational therapy, autism behavioral therapy, respite care, and access to recreational programs than their White counterparts.^18,19,20^ Similar differences in access to therapy have been observed for Asian children.^21,22^ These disparities may exist in part because parents of Black and Hispanic children with developmental disabilities report a lack of physician cultural sensitivity to their beliefs and values, not receiving enough time from physicians, lack of health information, and a poor sense of partnership in their child’s care.^21,23^ Among the numerous studies documenting racial and ethnic disparities in health care for children and youths with developmental disabilities, few name structural racism or discuss its role in driving racial disparities.^24,25,26,27^

Rethinking Whiteness as the norm for health in racial disparities research requires a deliberate approach, as has been proposed in antiracist health research models.^28,29,30,31,32^ One such model is the reproductive justice framework, a model that is used in sexual and reproductive health to center the voices of marginalized women and birthing people.^33^ Reproductive justice places an explicit emphasis on decentering Whiteness and addressing upstream factors that drive health disparities through dismantling racism (eg, policy, health care systems, clinicians, scientific research priorities).^33^ Although this framework has been used primarily for reproductive health research, the principle of decentering Whiteness as the standard for ideal health may be useful to operationalize in regard to other health conditions to challenge deficit-focused framings of the health of minoritized individuals. We operationalized the principle of decentering Whiteness from reproductive justice in an analysis of racial disparities in speech therapy receipt among children and youths with autism and other developmental disabilities from Southern California. The purpose of this study was to compare interpretation of 2 analyses of racial disparities among children and youths referred for speech therapy: a traditional, White-referenced analysis, and a Hispanic majority–referenced analysis.

## Methods

### Design and Setting

This cross-sectional study used referral and claims data from an integrated health system in Southern California from 2017 to 2020, where White-identified children and youths were a minority and Hispanic-identified children and youths were the majority. The study was approved by the institutional review boards at the University of California, Los Angeles and Kaiser Permanente Southern California; the same board granted a waiver of informed consent as this study was limited to analysis of deidentified administrative data.

### Conceptual Framework

Our analysis relied on the principle of decentering Whiteness from the reproductive justice framework. This model states that racism rather than race is the root cause of race-related health disparities and should be the target of interventions.^12^ Furthermore, it requires researchers to abandon traditional practices that uphold Whiteness as a health norm and perpetuate harm in minoritized communities.^33,34^ We applied the principle of decentering Whiteness by interpreting data on racial differences with 2 different lenses: (1) the traditional lens that holds the White racial group as the standard of health (ie, the reference category), and (2) the decentering Whiteness lens that centers the health of the majority, which in this study was Hispanic children and youths. Statistically, selecting the largest group as a reference category is often preferred. However, in racial disparities research, researchers frequently select White as the reference category even in studies where White individuals are a minority.^5,6,7^ Thus, we compared this traditional approach with a majority-referenced approach.

### Sample

This was a referral-level analysis. The sample of referrals of children and youths with developmental disabilities for speech therapy was derived from a patient registry at an integrated health system in Southern California. The registry contained 169 444 referrals for children, adolescents, and adults who were members of the health system to several types of habilitative therapy for speech and language delay, autism, or other developmental disabilities. Referrals were eligible for inclusion in the current analysis if the patient was under 27 years of age, had 1 or more diagnosed intellectual or developmental disability or delay, and received a referral for habilitative speech therapy, the most common type of therapy referral in the health system for this population. Some patients had multiple referrals to speech therapy within the same year. To prevent overestimation of effect size for children with multiple referrals, only the first referral of each year was included in the sample. The final sample was 66 402 referrals of children, adolescents, and transition-age youths referred for speech therapy (Figure).

**Figure.**
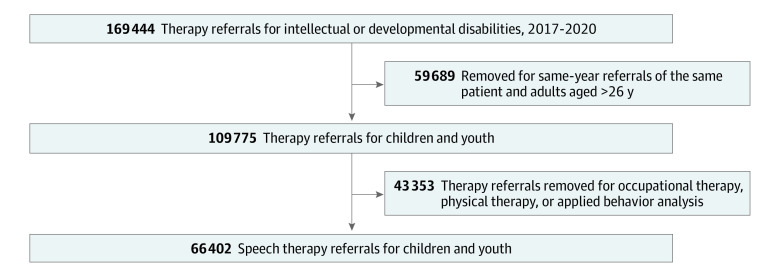
Study Flow Diagram

### Outcome

The primary outcome variable was whether children and youths received any speech therapy within a year of the initial referral. This dichotomous variable was determined from administrative claims data on whether speech therapy services were billed.

### Exposure

The primary exposure variable was child or youth race and ethnicity. Race was reported by parents or caregivers and included the following categories^35^: Asian, American Indian or Alaskan Native, Black and African American, Native Hawaiian or Pacific Islander, non-Hispanic White, or multiple races. Patients could also select other if they did not identify with any of these categories. Ethnicity included Hispanic or non-Hispanic; the race of anyone who selected Hispanic ethnicity was recoded to create a single race and ethnicity variable with all of the above race categories and an additional category for Hispanic and Latinx.^36^ In considering race, we assumed that race was both an individual sociocultural identity and a marker of a matrix of societal, historical, and political experiences that converge to create what we observe empirically as racial inequities, including structural racism.^12,13^

### Covariates

We examined other health and demographic factors to characterize the sample and adjust for confounding variables that might affect the association between the exposure and outcome. These were categorical variables for child or youth age (ages 0 to 3, 4 to 6, 7 to 12, 13 to 17, and 18 to 26 years), as service needs and recommendations vary by developmental stage^37,38^; sex, given that certain developmental disabilities affect more boys than girls^39^; primary referring disability diagnosis as level and urgency of need for speech therapy need may differ by diagnosis (autism spectrum disorder, cerebral palsy, developmental delay and intellectual disability, developmental motor function disorder, speech and language delay, feeding disorder, Down syndrome, other)^40^; insurance type, which may affect patients’ ability to access therapy via copays or limits for hours or visits (commercial, Medicaid, commercial and Medicaid, other)^41^; year of referral (2017 to 2020) to account for secular trends; and whether the patient had multiple referrals for speech therapy in their referral year, which might lead to overestimation of effect size.

### Statistical Analysis

A multivariable logistic regression model was estimated to examine the association between parent-identified race and ethnicity of the child or youth referred and odds of receipt of speech therapy after referral, adjusting for covariates (age, sex, insurance type, year, disability diagnosis, and multiple referrals). We calculated odds ratios from this model using 2 different reference categories, White (traditional reference category) and Hispanic (majority reference category). We also performed sensitivity analyses estimating the model with an interaction term for race and ethnicity and insurance, given known differences in insurance type by race and ethnicity.^42^ Analysis was conducted in R version 4.1.3 (R Project for Statistical Computing).

## Results

A total 66 402 referrals were included; 65 833 referrals (99.1%) were for children under age 17 years, 47 323 (71.3%) were for boys, and 39 959 (60.2%) were commercially insured. A majority of participants were identified as Hispanic (36705 [55.3%]); 6167 (9.3%) were identified as Asian, 4810 (7.2%) as Black, and 14 951 (22.5%) as White. Most referrals were for speech therapy for a speech or language delay (53 010 [79.8%]) or autism (10 975 [16.5%]) (Table 1). Eighty-one percent (54 156 referrals) resulted in receipt of speech therapy, and there were differences by race and ethnicity. Receipt of speech therapy occurred more frequency for children who were White (12 255 referrals [82.0%]), Asian (5080 referrals [82.4%]), and multiracial (518 referrals [84.5%]) compared with children who were Black (3858 referrals [80.2%]), Hispanic (29 979 referrals [81.7%]), American Indian or Alaskan Native (102 referrals [81.6%]), other (2085 referrals [78.6%]), or Pacific Islander (278 referrals [79.9%]). Unadjusted odds ratios (OR), using White as the reference category, indicated that children who identified their race as Black (OR, 0.89; 95% CI, 0.82-0.97) and other (OR, 0.81; 95% CI, 0.73-0.90) had lowered odds of speech therapy receipt.

**Table 1.** Sample Description

Sample characteristics	Referrals, No. (%) (N = 66 402)
Age, y	
0-3	31 589 (47.6)
4-6	21 120 (31.8)
7-12	10 880 (16.4)
13-17	2182 (3.3)
18-26	569 (0.9)
Sex	
Girls	19 079 (28.7)
Boys	47 323 (71.3)
Insurance type	
Both commercial and Medicaid	19 074 (28.7)
Commercial	39 959 (60.2)
Medicaid	1561 (2.4)
Other	5808 (8.7)
Race and ethnicity	
Asian	6167 (9.3)
Black and African American	4810 (7.2)
Hispanic and Latinx	36 705 (55.3)
Multiracial	613 (0.9)
American Indian or Alaskan Native	125 (0.2)
Other^a^	2651 (4.0)
Pacific Islander	348 (0.5)
White	14 951 (22.5)
Primary referring diagnosis	
Autism spectrum disorder	10 975 (16.5)
Cerebral palsy	12 (<0.1)
Developmental delay or intellectual disability	789 (1.2)
Developmental motor function disorder	23 (<0.1)
Down syndrome	106 (0.2)
Feeding disorder	81 (0.1)
Other	1406 (2.1)
Speech or language delay	53 010 (79.8)

^a^ Patients could self-identify as other if they chose not to identify as one of the listed racial or ethnic categories.

In the traditional approach to estimating racial disparities where the reference category was White, referrals had lower odds of resulting in actual receipt of speech therapy for children and youths who identified as Hispanic (aOR, 0.79; 95% CI, 0.75-0.83), Black (aOR, 0.72; 95% CI, 0.66-0.78), Pacific Islander (adjusted OR [aOR], 0.74; 95% CI, 0.57-0.98), and other (aOR, 0.85; 95% CI, 0.76-0.95) compared with referrals for White children and youths (Table 2). When using the majority race group (Hispanic) as the reference category to calculate odds ratios, referrals for children and youths who identified as White (aOR, 1.26; 95% CI, 1.20-1.30), Asian (aOR, 1.21; 95% CI, 1.12-1.30), and multiracial (aOR, 1.35; 95% CI, 1.08-1.71) had higher odds of resulting in actual service receipt in comparison with referrals for Hispanic children and youths. Odds of receipt of speech therapy after referral for children and youths who identified as Black remained lower in comparison with referrals of those who were Hispanic (aOR, 0.91; 95% CI, 0.84-0.98). Sensitivity analyses indicated there was a significant interaction between Black race and ethnicity and Medicaid (aOR, 0.77; 95% CI,0.63-0.94) (eTable in the Supplement). Black children with insurance types other than Medicaid also had lower odds of therapy receipt (aOR, 0.84; 95% CI, 0.75-0.94). Additionally, in the interacted model Asian children with no insurance had lower odds of speech therapy receipt (aOR, 0.67; 95% CI, 0.55-0.82).

**Table 2.** Odds of Speech Therapy Receipt After Referral by Child Race and Ethnicity^a^

Race and Ethnicity	OR (95% CI)	aOR (95% CI)
**White reference**		
Asian	1.03 (0.95-1.11)	0.96 (0.88-1.04)
Black	0.89 (0.82-0.97)	0.72 (0.66-0.78)
Hispanic	0.98 (0.93-1.03)	0.79 (0.75-0.83)
Multiple	1.20 (0.96-1.51)	1.07 (0.85-1.35)
American Indian or Alaskan Native	0.98 (0.63-1.57)	0.91 (0.57-1.5)
Other	0.81 (0.73-0.9)	0.85 (0.76-0.95)
Pacific Islander	0.87 (0.67-1.15)	0.74 (0.57-0.98)
**Hispanic reference**		
Asian	1.02 (0.97-1.07)	1.21 (1.12-1.3)
Black	1.05 (0.98-1.13)	0.91 (0.84-0.98)
Multiple	0.91 (0.84-0.98)	1.35 (1.08-1.71)
American Indian or Alaskan Native	1.22 (0.99-1.53)	1.15 (0.72-1.89)
Other	0.99 (0.64-1.6)	1.07 (0.97-1.19)
Pacific Islander	0.83 (0.75-0.91)	0.94 (0.72-1.24)
White	0.89 (0.69-1.17)	1.26 (1.2-1.33)

Abbreviations: aOR, adjusted odds ratio; OR, odds ratio.

^a^ The model is adjusted for age, sex, insurance type, primary referring diagnosis, and multiple referrals. A total 66 402 referrals were included of children ages 0 to 26 years for speech therapy for developmental disabilities, delays, and autism spectrum disorder from 2017 to 2020.

## Discussion

This analysis comparing White vs Hispanic reference categories to identify racial disparities in receipt of speech therapy after referral illustrates the potential value of identifying both racial disadvantage and racial advantage in health disparities research. Using multiple reference categories to identify unexplained racial advantage, in addition to disadvantage, may go beyond a deficits framework to illuminate health care structures that afford racial privilege to some children and youths over others. Previous studies examining the disparities in linkage to specialty services that affected Hispanic children had predominantly White samples with small percentages of Black or Hispanic children, which reinforces a White-centered approach to health and racialized framing of health deficits.^3,4,5^ Using multiple reference groups to analyze race disparities may result in greater sensitivity to health equity in interpretation of findings and additional intervention targets.

While integrated health care systems were designed to enhance access to care,^43^ children and youths receiving care within these systems are not immune to the effects of structural racism,^44^ as evidenced by a majority Hispanic patient population in this study receiving speech therapy at lower rates than White and Asian children and youths after referral. Both versions of the racial disparities model reported in this study are needed together to comprehensively identify unexplained racial advantage and disadvantage and to avoid obscuring disparities for children and youths who belong to minoritized racial and ethnic groups other than Black, including Hispanic children and youths who have experience significant gaps in behavioral health service access at a population level.^24,26,27^

Although Asian children and youths in this study had higher odds of receiving care after referral, these findings may not reflect the experiences of each Asian American subgroup. Despite Asian Americans being the fastest growing racial group in the US with diverse subgroups (including Chinese, Indian, Filipino, Vietnamese, Korean, Japanese, Pakistani, Cambodian, Hmong, Thai, Laotian, Bangladeshi, Burmese, Nepalese, Indonesian, Sri Lankan, Malaysian, Bhutanese, Mongolian, and others), researchers and health systems often aggregate Asians as one race, as was done in the health system under study,^45^ or even omit them entirely from samples.^46^ This practice masks the effects of structural racism and health inequities affecting Asian subgroups^9,47^; similar problems arise when Hispanic subgroups, including racial subgroups, are analyzed in a single category and within-group heterogeneity is obscured.^13,36^ Disaggregation of ethnic subgroups within broadly recognized racial and ethnic categories is an important future direction for health disparities research.

It is notable that Black children and youths had the lowest odds of receipt of speech therapy in relation to both White and Hispanic counterparts in adjusted and unadjusted models, regardless of insurance type. This finding may be driven by structural racism in health care systems as well as efforts by Black parents to protect their children from racism. Qualitative studies have found that some Black families intentionally delay treatment or diagnosis of developmental disabilities and encourage “normal” development as a way to protect their children from unequal treatment, stereotyping, and discrimination.^48^ Out of a lack of structural and cultural competence,^49^ clinicians in turn may understate the value and importance of speech therapy for their Black patients because of incorrect assumptions or stereotypes that Black parents will not pursue speech therapy.^50,51^ Lowered odds of receipt of speech therapy among Black children and youths may also be driven by structural racism in health care systems, including divestment of health care resources and infrastructure from Black communities and substandard or discriminatory care.^4^ After referral, Black families may experience fewer local health care options and need to traverse longer distances outside of their communities to receive care.^52^ Clinicians may have inflexible and inconvenient hours at odds with parental workdays or may lack community or cultural knowledge.^15,20^ In these ways, structural racism upholds the litany of barriers to successful receipt of care in Black populations, which consequently concentrates advantage in White populations.

One challenge researchers may encounter in using multiple reference groups in racial disparities research is that individuals who identify as White are the majority in many research samples. This may result in a de facto statistical decision to use White race as the reference group, despite known heterogeneity in health outcomes among these individuals.^9^ Although the non-White population share is growing in the US, there is a long history of exclusion of people of other racial and ethnic groups in biomedical and public health research, leading to overrepresentation of White participants in studies.^53^ The use of White as a de facto reference group may also be due to implicit bias from scientists, who are themselves predominantly White.^54^ To ensure the ability to make comparisons of racial disparities across multiple racial and ethnic groups, researchers may consider multisite studies with different population demographics from which samples can be drawn, oversampling of racial and ethnic minority groups, and community engagement to increase the participation of marginalized populations in research. Researchers may also consider using absolute rather than relative measures in racial disparities research to avoid inadvertent elevation of the White race as the standard for health. Given that multiple studies of racial disparities use White as the reference even when White individuals are a minority in the sample,^5,6,7,8^ researchers may consider calculating the racial disparities against at least 2 reference groups selected on the basis of community context and comparable structural advantage or structural disadvantage experienced between different racial groups.^55^

Researchers should be intentional about defining the conceptual rationale for investigating race and ethnicity in health research, as racism rather than race itself is an underlying driver of health disparities that is not often discussed explicitly in research reports.^12,13,56^ When race and ethnicity is studied as a main exposure with adjustment for potential confounders, it is important to have a conceptual rationale for the role of covariates that are downstream of race and racism (eg, insurance type, income, level of education, neighborhood), which may have a mediating role in determining outcomes.^57,58^ Identifying moderating or mediating influences on health disparities may allow for targeting modifiable factors with interventions. When race and ethnicity are conceptualized as a protentional confounding factor, researchers may consider stratifying analyses by subgroups rather than simply adjusting for racial differences.

### Strengths and Limitations

The strengths of this study include its large, diverse sample of referrals of children and youths from a Southern California health system. We borrowed from an established racial equity framework (ie, reproductive justice) to inform an analysis of racial disparities.

Limitations of the study include lack of disaggregated subdata on racial groups (eg, Asian subgroups), the cross-sectional nature of the study, a lack of detailed information on family and neighborhood context, and no measures of perceived interpersonal or intrapersonal discrimination. Using administrative and billing data might introduce risk for errors in cases where billing codes were miscoded, and the referral-level nature of the sample does not allow for identification of duration of therapy. Although our findings suggest that there is structural racism embedded in health systems, the patient-level data used in this study cannot identify specific system-level manifestations of structural racism. Future studies should investigate health system and community measures of structural racism and their role in observed health care disparities for children and youths with developmental disabilities.

## Conclusions

In this study of racial disparities in referrals for and use of speech therapy among children and youths with developmental disabilities, using Hispanic children and youth as the reference group found advantage for White, Asian, and multiracial individuals and persistent disadvantage for Black individuals. Our study demonstrates the value of decentering Whiteness in interpreting racial disparities research and considering racial differences against multiple referents. Racial disparities researchers should consider investigating multiple between-group differences instead of exclusively using White as the default reference category, which may obscure unexplained White advantage embedded in health systems and reinforce problematic framings of Black families. Racial disparities in receipt of disability services among children and youths with developmental disabilities may be driven by structural racism that disproportionately harms Black children and youths and disproportionately advantages those who are White and Asian. By using multiple reference groups and informing analyses with racial equity frameworks, researchers can evaluate findings in terms of both unexplained racial disadvantage and advantage and target interventions toward both problems.

## Notes

Supplement. **eTable.** Odds of Speech Therapy Receipt by Child Race/Ethnicity and Insurance Type
