# Supplementary material for: Cross-sectional Comparison of Disparities by Race Using White vs Hispanic as Reference Among Children and Youths With Developmental Disabilities Referred for Speech Therapy
Source: JAMA Netw Open. 2022 Oct 4;5(10):e2234453. doi: 10.1001/jamanetworkopen.2022.34453 (PMC9533182; doi:10.1001/jamanetworkopen.2022.34453)
Supplement: Supplement. — eTable. Odds of Speech Therapy Receipt by Child Race/Ethnicity and Insurance Type [file jamanetwopen-e2234453-s001.pdf]

Supplemental Online Content

Elliott T, Floyd James K, Coleman KJ, Skrine Jeffers K, Nau CL, Choi K. Cross-sectional comparison of disparities by race using White vs Hispanic as reference among children and youths with developmental disabilities referred for speech therapy. *JAMA Netw Open.* 2022;5(10):e2234453. doi:10.1001/jamanetworkopen.2022.34453

eTable. Odds of Speech Therapy Receipt by Child Race/Ethnicity and Insurance Type

This supplemental material has been provided by the authors to give readers additional information about their work.

© 2022 Elliott T et al. *JAMA Network Open.*

eTable. **Odds of Speech Therapy Receipt by Child Race/Ethnicity and Insurance Type**

Odds ratios with White reference category	OR	95% CI (lower bound)	95% CI (upper bound)
**Race/ethnicity (reference: White)**			
Asian	1.03	0.93	1.14
Black	0.84	0.75	0.94
Hispanic	0.80	0.75	0.85
Multiple	1.11	0.84	1.49
Native American/Alaskan	1.00	0.55	1.98
Other	0.89	0.79	1.02
Pacific Islander	0.74	0.55	1.03
**Insurance type (reference: Commercial)**			
Commercial and Medicaid	1.47	1.02	2.21
Medicaid	1.22	1.07	1.38
None	0.39	0.35	0.43
**Interactions**			
Race/ethnicity*Commercial and Medicaid			
Asian*Commercial and Medicaid	0.99	0.51	2.01
Black*Commercial and Medicaid	0.59	0.33	1.07
Hispanic*Commercial and Medicaid	0.80	0.51	1.21
Multiple*Commercial and Medicaid	>100.0	8.46	NA
Native American/Alaskan*Commercial and Medicaid	NA	NA	NA
Other*Commercial and Medicaid	1.28	0.33	8.40
Pacific Islander*Commercial and Medicaid	0.12	0.00	3.17
Race/ethnicity*Medicaid			
Asian*Medicaid	1.12	0.86	1.47
Black*Medicaid	0.77	0.63	0.94
Hispanic*Medicaid	1.09	0.95	1.25
Multiple*Medicaid	0.83	0.45	1.61
Native American/Alaskan*Medicaid	1.30	0.37	6.13
Other*Medicaid	0.62	0.44	0.87
Pacific Islander*Medicaid	1.50	0.67	3.85
Race/ethnicity*None			
Asian*None	0.67	0.55	0.82
Black*None	0.73	0.53	0.99
Hispanic*None	0.88	0.76	1.02
Multiple*None	0.91	0.49	1.73
Native American/Alaskan*None	0.51	0.14	1.74
Other*None	1.00	0.76	1.32
Pacific Islander*None	0.80	0.32	2.10

© 2022 Elliott T et al. *JAMA Network Open.*

Odds ratios with Hispanic as reference category	OR	95% CI (lower bound)	95% CI (upper bound)
**Race/ethnicity (reference: Hispanic)**			
Asian	1.29	1.18	1.41
Black	1.05	0.94	1.17
Multiple	1.39	1.06	1.86
Native American/Alaskan	1.25	0.69	2.48
Other	1.12	0.99	1.27
Pacific Islander	0.93	0.68	1.28
White	1.25	1.17	1.34
**Insurance type (reference: Commercial)**			
Commercial and Medicaid	1.17	0.97	1.42
Medicaid	1.33	1.25	1.41
None	0.34	0.31	0.37
**Interactions**			
Race/ethnicity*Commercial and Medicaid			
Asian*Commercial and Medicaid	1.24	0.71	2.34
Black*Commercial and Medicaid	0.75	0.47	1.23
Multiple*Commercial and Medicaid	>100.0	6.92	NA
Native American/Alaskan*Commercial and Medicaid	NA	NA	NA
Other*Commercial and Medicaid	1.60	0.44	10.31
Pacific Islander*Commercial and Medicaid	0.15	0.01	3.93
White*Commercial and Medicaid	1.26	0.83	1.96
Race/ethnicity*Medicaid			
Asian*Medicaid	1.03	0.81	1.32
Black*Medicaid	0.70	0.59	0.83
Multiple*Medicaid	0.76	0.41	1.46
Native American/Alaskan*Medicaid	1.19	0.34	5.61
Other*Medicaid	0.57	0.42	0.79
Pacific Islander*Medicaid	1.37	0.62	3.51
White*Medicaid	0.92	0.80	1.06
Race/ethnicity*None			
Asian*None	0.76	0.63	0.93
Black*None	0.82	0.61	1.12
Multiple*None	1.03	0.56	1.95
Native American/Alaskan*None	0.58	0.16	1.97
Other*None	1.13	0.86	1.49
Pacific Islander*None	0.91	0.36	2.38
White*None	1.13	0.98	1.31

Notes. Multiple logistic regression model estimating odds of receipt of speech therapy after referral by race/ethnicity, showing odds ratios with White as the reference category versus Hispanic as the reference category. The model is are adjusted for age, gender, primary referring diagnosis, and multiple referrals. N=66402 referrals of children ages 0 to 26 for speech therapy for developmental disabilities, delays, and/or autism spectrum disorder from 2017 to 2020.

© 2022 Elliott T et al. *JAMA Network Open.*
